# Developmental Lead Exposure Induces Tactile Defensiveness in Rhesus Monkeys (*Macaca Mulatta*)

**DOI:** 10.1289/ehp.11203

**Published:** 2008-05-30

**Authors:** Colleen F. Moore, Lisa L. Gajewski, Nellie K. Laughlin, Melissa L. Luck, Julie A. Larson, Mary L. Schneider

**Affiliations:** 1 Department of Psychology; 2 Harlow Center for Biological Psychology; 3 Department of Kinesiology, University of Wisconsin-Madison, Madison, Wisconsin, USA; 4 Psychology Department, Marquette University, Milwaukee, Wisconsin, USA

**Keywords:** lead exposure, sensory processing disorder, succimer chelation, tactile defensiveness, withdrawal response

## Abstract

**Background:**

Tactile defensiveness in children is associated with difficult social relations, emotional dysregulation, and inattention. However, there are no studies of lead exposure and tactile defensiveness in children or animals in spite of the fact that lead exposure is also associated with inattention and emotional dysregulation.

**Objectives:**

In this study we tested whether lead exposure induces tactile defensiveness in rhesus monkeys.

**Methods:**

We tested 61 monkeys from a 3 (no lead, 1-year lead, 2-year lead) × 2 (succimer chelation or not) factorial experiment for tactile defensiveness at 4 years of age. Lead-treated monkeys had been orally administered lead in a daily milk solution from 8 days of life to either 1 or 2 years of age to produce blood lead levels of 35–40 mg/dL. Succimer chelation therapy or placebo was administered at 1 year of age. We measured tactile defensiveness using six repeated trials of each of three textures as a swipe to the cheek and neck.

**Results:**

Lead-exposed monkeys showed higher negative responses to repeated tactile stimulation compared with controls. Blood lead during the first 3 months of life was positively correlated with the negative response on the tactile defensiveness test. There was an interaction of lead exposure × succimer chelation × trials, but it is not clear that succimer chelation was beneficial with respect to tactile defensiveness.

**Conclusions:**

This is the first report to implicate lead as a potential cause of tactile defensiveness. Research should examine whether lead exposure is associated with tactile defensiveness in children.

Exposure to lead, a pervasive environmental pollutant, is associated with numerous adverse developmental effects including impairments in cognitive function, behavioral problems, and sensory abnormalities. In this study we used a nonhuman primate model to demonstrate for the first time that lead can induce tactile defensiveness, a form of sensory processing disorder. We have previously reported that prenatal stress and prenatal alcohol exposure induced tactile defensiveness in monkey offspring (Schneider et al. 2008).

“Tactile defensiveness” is a term first used by A. Jean Ayres, an occupational therapist and psychologist, to describe a subtype of sensory processing disorder that involves “feelings of discomfort and a desire to escape the situation when certain types of tactile stimuli are experienced” (Ayres 1964). Children with tactile defensiveness are more likely to show exaggerated or otherwise unusual responses to typically neutral tactile stimuli. The disorder has been linked to hyperactivity and distractibility, as well as to academic learning problems, in children (Ayres 1972; Mulligan 1998). Children with tactile defensiveness may have difficulties with social relationships because avoidance of touch may offend friends and relatives. Children may be considered to be demonstrating “poor behavior” when interactions with peers and family members involving touch induce anger or negative emotional responses. Recent interest in tactile defensiveness is due partly to the rise in the rate of diagnosis of autism, because abnormal sensory features are common but not universal in children with autism. Baranek et al. (2006) reported that, compared with typically developing children, 69% of children with autism had elevated sensory symptoms. These sensory symptoms included hyperresponsiveness (exaggerated behavioral responses to sensory stimuli including touch avoidance) and hyporesponsiveness, including reduced response to pain (Baranek et al. 2006, 2007). Although the causes remain elusive, tactile defensiveness is thought to reflect fundamental aberrations in arousal and sensory gating mechanisms that have cascading effects on other functions (Lane 2002).

Early environmental factors clearly play a role in sensory processing disorders and probably interact with genetic factors as well. Children who spent time in Eastern European orphanages exhibited a higher rate of sensory processing disorders, including tactile defensiveness, and a longer length of institutionalization was associated with more atypical sensory regulation (Lin et al. 2005). Boys with attention deficit hyperactivity disorder (ADHD) also showed a higher rate of tactile defensiveness than did a comparison sample without ADHD (Parush et al. 2007). In a recent study of > 1,000 twins, Goldsmith et al. (2006) found that tactile and auditory defensiveness showed moderate genetic influences. Studies of the pattern of electrodermal responses to repeated sensory stimuli showed that individuals with the fragile *X* mutation failed to habituate to repeated sensory stimuli, while controls showed the expected habituation pattern (Miller et al. 1999). Children with sensory processing disorder also demonstrated less auditory sensory gating than typically developing children as measured by event-related potential (Davies and Gavin 2007). Reduced sensory gating might be related to some of the reported behavioral characteristics of children with sensory processing disorder, including inattention, impulsivity, hyperactivity, emotional lability and disorganization (Miller et al. 2001).

A large body of knowledge indicates that childhood lead exposure is associated with impaired cognitive performance and behavioral problems including hyperactive and impulsive behaviors, inattention, fear, withdrawal behaviors, and juvenile delinquency (Bellinger et al. 1994; Braun et al. 2006; Chiodo et al. 2007; Dietrich et al. 2001; Fergusson et al. 1988; Needleman et al. 1996, 2002). Some of these effects of lead—in particular inattention, hyperactivity, and poor cognitive performance—overlap with conditions found in children with tactile defensiveness. Hearing thresholds, visual evoked potentials, and postural balance are also adversely affected by lead (Bhattacharya et al. 2007; Chuang et al. 2007). Because even the best human studies cannot definitively establish causality and because lead policies have been surrounded by controversies [for an overview, see Moore (2003)], animal models are especially important. Studies with rhesus monkeys have established that lead exposure can cause adverse developmental effects such as impaired early neurobehavioral development, impaired reversal learning performance, reduced selective attention and association processes, sensory abnormalities, and increased activity and exploration in an open field area (Bushnell et al. 1977; Ferguson et al. 1996; Lasky and Laughlin, 2001; Lasky et al. 1995, 2001a; Laughlin et al. 1983, 1999; Levin et al. 1988).

In recent pediatric clinical trials, chelation with succimer failed to reduce the negative impacts of lead exposure (Dietrich et al. 2004). However, in an earlier study Ruff et al. (1993) found that improvements in cognitive scores were correlated with declines in blood lead, regardless of chelation treatment. Recent research with rats has shown that chelation with succimer can lessen the exaggerated emotional reactions to errors and reward omissions induced by lead exposure (Beaudin et al. 2007). The same study also found that control animals given succimer showed stronger reactions to errors than placebo controls. Another study showed cognitive impairment in rats as a result of succimer chelation without exposure to lead, but chelation improved attention and cognitive function in rats that were exposed to lead (Stangle et al. 2007). Therefore, succimer chelation may provide both benefits and risks. In this article we report the results of a test for tactile defensiveness that was administered to monkeys from a longitudinal experiment that independently manipulated lead exposure and succimer chelation.

## Methods

Test subjects were 61 female rhesus monkeys (*Macaca mulatta*) derived from the colony of the University of Wisconsin Harlow Center for Biological Psychology. The study monkeys were randomly assigned at birth to one of six treatment groups in a factorial design with three levels of lead exposure (0, 1, or 2 years of lead intake) and two levels of chelation treatment (placebo and succimer). All procedures were approved by the University of Wisconsin-Madison Animal Care and Use Committee. The project originally involved 72 animals. At the time of this study, 66 animals were available for testing, as reported previously (Lasky et al. 2001b). Two animals (a 1-year lead chelated animal and a control chelated animal) were not tested in the present study because they succeeded in grabbing and chewing or eating the first tactile stimulus early in the session. Three animals (a 1-year lead nonchelated, a control nonchelated, and a 1-year lead chelated animal) were mistakenly omitted because they were not listed on the roster used to identify animals for testing in this study. All of the other monkeys that were available from the project were successfully tested: 11 control nonchelated, 11 control chelated, 8 1-year lead nonchelated, 11 1-year lead chelated, 10 2-year lead nonchelated, and 10 2-year lead chelated. The mean ages of the animals at sensory testing were 4.14, 4.10, 4.01, 4.11, 4.10, and 4.29 years in the six groups, respectively. The age range was 2.98–5.27 years. There were no age differences among conditions in age at time of testing (*p* > 0.10).

### Lead dosing

Lead-exposed monkeys were administered lead from day 8 of life to either 1 or 2 years of age at levels to produce blood lead levels of 35–40 μg/dL. Even when exposure is standard across animals, there are still individual differences in blood lead concentration (Laughlin 1995). In the present study we titrated the lead exposures individually to reach target blood lead values. The target value of 35–40 μg/dL was chosen because it is within the range of blood lead levels in children participating in pediatric clinical trials [Treatment of Lead-exposed Children Trial Group (TLC) 1998]. Lead was given as a solution of lead acetate/50% glucose in 4 cc of commercial milk formula (Similac with Iron; Ross Products Division, Abbott Laboratories, Columbus, OH). The solution was delivered directly into the mouth using a 5-cc syringe while the infant was attached to the mother. All mothers wore permanent collars. The mother’s collar was briefly attached to the cage to allow access to the infant without removing the infant from the mother during lead or placebo dosing. Controls were treated identically except that the milk formula contained distilled water in a volume equal to the lead acetate/glucose solution. Postweaning, the solution was administered in the home cage with the same lead acetate/glucose mixture diluted with apple juice or fruit-flavored drink. Additional details of the lead dosing procedure have been reported elsewhere (Lasky et al. 2001b).

### Chelation therapy

Succimer treatment was administered following standard clinical procedures (Graziano et al. 1992). Succimer was removed from the capsules (Chemet; Sanofi Winthrop, New York, NY), dissolved in a syringe with apple juice and administered to the monkey within 15 min of being dissolved. Succimer was administered at a dose of 30 mg/kg/day divided into three doses (at 0900, 1600, and 2300 hours) for a total treatment regime of 19 consecutive days. Placebo capsules obtained from the Treatment of Lead-exposed Children clinical trial (TLC 1998) were administered identically. The first chelation regime began at 53 weeks of age (chelation 1) commensurate with the termination of lead intake for the 1-year lead-exposed group and controls. The second chelation therapy began at 65 weeks of age (chelation 2), 9 weeks after completion of the first chelation. All subjects (including controls) were housed in metabolic cages for the first 5 days of each chelation regime. Lead and lead-vehicle dosing were discontinued during the 19 days of each chelation. All lead dosing and chelation therapy were administered “blind” to the treatment condition of the subject.

### Blood sampling

We obtained blood samples from all infant monkeys at week 1 postpartum before the onset of dosing. Thereafter, blood samples were obtained from all monkeys (including controls) every other week beginning at either week 3 or 4. Blood samples were always collected before lead dosing on any given day. For sampling, approximately 2 cc of blood was collected by femoral venipuncture into a 2-cc evacuated collection tube (Beckton Dickenson, Franklin Lakes, NJ) containing 48 mL EDTA. The samples were refrigerated immediately after collection (5°C) and stored at −20°C as whole blood until assayed for lead concentration. Blood lead levels were determined by the Wisconsin State Laboratory of Hygiene (Madison, WI) using electrothermal atomic absorption spectrophotometry with Zeeman background correction (Hitachi Instruments, San Jose, CA). Details of the blood lead analysis and the biweekly mean blood lead values are reported elsewhere (Lasky et al. 2001b). Because blood lead levels are well characterized for control infants in our laboratory, samples from all control infants were analyzed at week 1 but only periodically thereafter to identify inadvertent lead exposure. Although we were not able to assess blood lead concentrations concurrently with the administration of the tactile defensiveness assessment for the present study, the blood lead values of all monkeys had stabilized below the assay limit of 5 μg/dL prior to the study.

### Sensory Processing Scale for Monkeys (SPS-M)

The SPS-M (Schneider et al. 2008) was developed by adapting procedures from laboratory observational measures of sensory processing for children (Baranek and Berkson 1994; Miller et al. 1999). Correlations between parental reports and the laboratory observational measures of sensory hyperresponsiveness (including tactile defensiveness) have been modest (correlations of 0.20 to 0.40) (Baranek and Berkson 1994). The SPS-M has been used in a previous study of the effects of moderate level fetal alcohol exposure, alone or in conjunction with pre-natal stress (Schneider et al. 2008). In the present study, sensory processing testing followed the same procedures as described by Schneider et al. (2008). Testing was conducted in a 53 × 44 cm testing cage with vertical bars spaced 5.5 cm apart. The cage was situated in a dimly lit and sound-shielded room (62 dB) with a masking white noise of 65–70 dB. Each monkey was tested individually by a human experimenter who stood beside the cage and administered the tactile items through the bars of the cage. A second experimenter videotaped the session for later scoring. Both experimenters were blind to the experimental conditions of the animals and unfamiliar to the animals.

The first tactile stimulus consisted of a 12.5-cm feather, which delivered light tactile stimulation. The second stimulus, a 7-cm cotton ball, delivered a soft but slightly firmer tactile stimulation. Finally, the third stimulus, a 15-cm stiff craft brush, delivered a scratchy but innocuous tactile stimulation. All stimuli were attached to a 91-cm dowel so the experimenter could maintain a safe distance from the monkey’s cage. Six trials of each stimulus were administered in an invariant order, as listed above, as a swipe to the cheek and neck area. Before the first presentation of each stimulus, the stimulus was placed in full view and in touching range of the monkey and remained there for approximately 3 sec. Stimuli were then applied repeatedly to the same side of the animal for approximately 2 sec per trial, with an intertrial interval of approximately 2 sec and a pause between each of the textures of approximately 4 sec. The testing session lasted for approximately 10 min. Raters blind to the treatment conditions of the animals scored the videotapes. Each of 18 trials was scored for degree of withdrawal and negative reaction to the tactile stimulus using a 0–3 rating scale in 0.25 increments, with the integers labeled as follows: 0 = no withdrawal; 1 = slight withdrawal, such as turning head away from the stimulation; 2 = moderate withdrawal, such as turning full body away from stimulation; and 3 = extreme withdrawal, such as moving body away from stimulation. Inter-rater reliability, as the percentage agreement within ± 0.25 on the rating scale, exceeded 99%. Prior to this sensory testing, the animals had participated in a variety of other behavioral tests. All animals underwent the identical protocols, including behavioral assessments of neonatal development and growth (Laughlin et al. 1999), weaning at 6 months of age according to typical Harlow Primate Laboratory procedures, identical social housing thereafter (5 females, 1 male), open field testing during infancy (Lasky and Laughlin 2001), auditory function assessment (Lasky et al. 2001b), and learning tasks.

### Statistical analysis

The rated response on each trial was the dependent variable in a lead exposure (0, 1, or 2 years) × chelation (placebo or succimer) × texture (feather, cotton, brush) × trial (6) analysis of variance (ANOVA) with repeated measures on the last two factors. We used the Huyhn-Feldt adjustment of *p*-values to adjust for possible violations of the sphericity assumption for effects involving repeated measures. Post hoc tests were conducted using the Tukey–Kramer method (Keppel and Wickens 2004). In addition, we calculated each animal’s overall magnitude of response as the mean response over all trials. An index of habituation was calculated for the six trials of each texture by using linear trend coefficients (Keppel and Wickens 2004) and then averaging over textures. To examine the relationships of sensory testing scores to blood lead levels during different developmental periods, we averaged biweekly blood lead concentrations over different epochs: very early (weeks 2–6), early (weeks 2–12), preweaning (weeks 14–26), 6–12 months (weeks 28–52), and postchelation (weeks 68–112). The mean blood lead values were all correlated > 0.9 with area under the curve calculated by the trapezoid method using the blood lead concentrations as the *y* values and the biweekly test period as the *x* values.

## Results

### Treatment effects on sensory response scores

**Figure 1:**
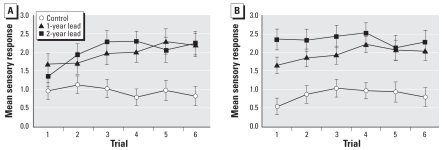
Mean sensory response scores as a function of trials and lead treatment in nonchelated (*A*) and chelated (*B*) animals. Error bars indicate ± 1 SE.

The mean sensory response scores over trials of the six treatment groups are presented in Figure 1. The overall ANOVA yielded significant main effects of lead exposure [*F*(2, 55) = 14.13; *p* < 0.001], texture [*F*(2, 110) = 4.66; *p* < 0.02], and trials [*F*(5, 27) = 6.90; *p* < 0.001], and interactions of lead × trial [*F*(10, 275) = 2.54; *p* < 0.01] and lead × chelation × trial [*F*(10, 275) = 2.30; *p* < 0.02].

The most striking finding is that the sensory scores of animals exposed to lead were uniformly higher than the scores of animals not exposed to lead [mean ± SE = 1.83 ± 0.16, 1.99 ± 0.17, and 0.91 ± 0.15, for 2-year lead, 1-year lead, and no-lead exposure groups, respectively. Post hoc tests showed that the no-lead condition differed significantly from the 1-year and the 2-year lead conditions (*p*-values < 0.001) but that the two lead exposure conditions did not differ (*p* > 0.20). The feather and brush created the highest overall negative sensory response, and cotton yielded the lowest overall response (mean ± SE = 1.61 ± 0.09, 1.44 ± 0.11, and 1.67 ± 0.11, for feather, cotton, and brush, respectively). There was slight sensitization over trials (significant main effect of trials), and the magnitude of the sensitization effect was greater for lead-exposed animals than controls (significant lead × trial interaction).

Chelation therapy did slightly alter the pattern of response over trials as indicated by the significant lead × chelation × trial interaction (Figure 1). To clarify the interaction, we also separately tested the differences among the six treatment groups at the first trial and at the last trial. For the first trial we found a significant effect of lead-exposure condition [*F*(2, 55) = 9.78; *p* < 0.001] and a condition × chelation effect [*F*(2, 55) = 3.98; *p* < 0.03]. For the last trial we observed a significant effect of lead-exposure condition [*F*(2, 55) = 15.03; *p* < 0.0001] but not a condition × chelation interaction (*p* > 0.20). On the first trial, the control nonchelated group scored significantly lower than the 2-year lead chelated group (*p* < 0.05) but did not differ from any of the other groups in the post hoc tests. The control chelated group scored significantly lower than the 1-year lead nonchelated, 1-year lead chelated, and 2-year lead chelated groups (*p* < 0.05) but was not significantly different from the 2-year lead nonchelated group or the control nonchelated group (*p* > 0.20). The 2-year lead nonchelated group scored slightly lower than the 2-year lead chelated group (*p* < 0.10). On the last trial, both control groups scored significantly lower than all four lead-exposed groups (*p*-values < 0.05), but they were not statistically different from each other (*p* > 0.20). None of the lead-exposed groups differed significantly from each other (*p*-values > 0.20).

### Correlations of sensory scores with blood lead concentrations

Table 1 presents the correlations between the sensory test summary scores and the mean blood lead concentrations across different periods of development. These correlations are based on the lead-exposed animals only. The mean blood lead concentrations for the different periods of development are presented in Table 2. For the first year of life, we found no differences in mean blood lead concentrations among the lead-exposed treatment groups.

**Figure 2:**
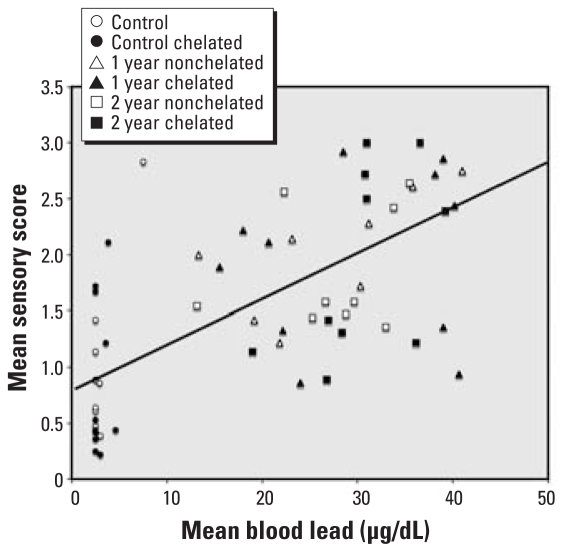
Scatterplot of mean blood lead concentrations (μg/dL) at 2–12 weeks of age and mean sensory test score for lead-treated animals (*n* = 39). The solid line shows the regression of lead (*x*) and sensory score (*y*): *y* = 0.82 + 0.00375*x; r* = 0.42; *p* < 0.01. Control animals (*n* = 22) are presented for comparison but were not included in calculating the regression line. Including controls, *r* = 0.62, *p* < 0.01.

As shown in Table 1, early blood lead concentration was positively correlated with the magnitude of negative response on the sensory test, with the strongest relationship found for lead exposure at 2–12 weeks of age. By 6–12 months of age (postweaning), the correlation was somewhat weaker, and the sign of the correlation between the magnitude of response on the sensory test and blood lead concentration was negative for the postchelation period of the study. Figure 2 presents a scatterplot of the relationship between blood lead level from weeks 2–12 postpartum and the sensory test summary score. Examination of the correlations separately by lead treatment groups and by chelation group showed that the correlations within groups were similar to the overall pattern. We found no significant correlations between blood lead concentrations and habituation scores.

## Discussion

There were two principal findings in the present study. First, lead-exposed monkeys showed significantly more negative responses to repeated tactile stimuli compared with monkeys not exposed to lead. Second, lead exposure measured during early life (first 3 months) was positively correlated with the magnitude of the negative response (i.e., the degree of tactile defensiveness).

Our first finding, that early postnatal lead exposure induced a more negative response to tactile stimuli, implicates early lead exposure as a possible cause of tactile defensiveness. Conceptually, sensory defensiveness is an alteration in the ability to accommodate to novel stimulation, an aspect of behavioral regulation in children. Although sensory defensiveness has not been evaluated in lead-exposed children, many of the cognitive, behavioral, and social problems associated with early lead exposure in children could be linked to sensory defensiveness. Hence, our findings provide experimental evidence relevant to the large literature in children suggesting that postnatal lead exposure may be associated with long-lasting effects on attention and aspects of behavioral regulation (Braun et al. 2006; Chiodo et al. 2007; Dietrich et al. 2001; Needleman et al. 1996, 2002).

The mechanisms underlying lead effects on behavior regulation, however, have not been clearly delineated. Rodent studies suggest that the hippocampus is important for emotion regulation and accommodation to novelty. Lead is a potent inhibitor of the *N*-methyl-d-aspartate (NMDA) subtype of excitatory amino acid receptors in the hippocampus (Guilarte and Miceli 1992). Activation of the NMDA receptor subtype is considered critical to long-term potentiation, that is, long-lasting alterations at the synapse that facilitate neuronal communication are involved in learning and memory (Altmann et al. 1991). In addition, chronic exposure to lead in rats has also been shown to decrease the survival of newly born granule cells in the CA3 region of the hippocampus and to result in reduced dendritic branching (Verina et al. 2007). Because approximately 85% of granule neurons of the dentate gyrus of the hippocampus are produced postnatally in rodents and neurogenesis continues throughout the life span (Hastings et al. 2001), lead-induced reductions in hippocampal neurogenesis and altered dendritic morphology might also be the foundation for the reduced hippocampal neuronal plasticity believed to underlie altered accommodation to novelty.

Our second finding was that blood lead concentration during early life (first 3 months) was more strongly correlated with the magnitude of the negative response to tactile stimulation than was later blood lead concentration. Rakic (1988) described three broad phases of brain development in rhesus monkeys: generation of neurons, neuronal migration, and synaptogenesis. The phase of rapid synaptogenesis, which occurs synchronously in the somatosensory, motor, and association areas, begins at gestation day 112 and continues to the third month postnatally (Bourgeois and Rakic 1993). Therefore, our findings suggest that the period of rapid synaptogenesis in the monkey is a time of enhanced vulnerability to lead exposure for the appearance of later impairments in sensory regulation. The period of most rapid synaptogenesis in children appears to extend from approximately a few weeks before birth to 4 years of age, depending on the particular brain region (Huttenlocher 2002). The peak period of lead exposure for children is at approximately 2 years of age and is likely to be concurrent with the period of rapid synaptogenesis.

In addition to these two findings, chelation with succimer had a small but significant effect, mainly on initial responses to the tactile stimuli. On the first trial, chelation slightly lowered the tactile defensiveness of the control group but slightly exacerbated the tactile defensiveness of the 2-year lead-exposed group. The lower response on the first trial in the control group should not necessarily be interpreted as a salutary effect of chelation, because it was not significantly different from the nonchelated control group. Despite the overall strong effects of postnatal lead exposure in inducing tactile defensiveness, on the last trial of tactile stimulation chelation had no significant effect on the response of the lead-exposed animals. Therefore, the present study does not provide a clear indication of whether or not succimer chelation for lead exposure is beneficial with respect to tactile defensiveness. Chelation was administered at 1 year of age, and the correlation of tactile defensiveness with blood lead concentration in the second year of life was nonsignificant. This contrasts with the finding that blood lead levels early in life (first 3 months) were significantly predictive of tactile defensiveness.

In the TLC study of the effects of succimer chelation on lead-exposed children, the placebo group performed significantly better on the attention and executive function tasks, tasks that require good sensory gating (Dietrich et al. 2004). Overall, the results on 4 of 12 tests in the TLC study favored the placebo group, in spite of the fact that higher lead at the time of testing was associated with lower full-scale IQ test scores, lower reading scores, and higher externalizing and school problems.

Our results show that lead exposure can induce increased negative response to repeated tactile stimulation, the phenomenon termed “tactile defensiveness.” These results have important implications for children, given that tactile defensiveness can have profound adverse effects on a child’s successful participation in school, home, and community. In the present study, as well as in other experimental models using animal subjects (Beaudin 2007), chelation therapy slightly altered the patterns of behavior produced by lead exposure. In the present study, it is not clear whether or not chelation was helpful. The finding that tactile defensiveness was significantly correlated with early life blood lead concentrations in these animals, combined with the TLC findings that succimer was not beneficial, punctuate the importance of protecting children from lead exposure rather than relying on later treatments to remove lead from tissues. The greater effectiveness and fewer side effects of succimer do make it potentially more desirable than other chelators. Future research might examine the effectiveness of succimer administered earlier in life than in the present study, perhaps before the end of the period of rapid synaptogenesis.

Because tactile defensiveness is thought to depend on fundamental processes of sensory gating that may influence a wide range of behavior, the results of the present study suggest that it is important to examine the relationship between lead exposure and tactile defensiveness in children. Research is also needed to better understand the neural mechanisms underlying the lead-induced tactile defensiveness found here. An additional challenge will be to determine whether remediation of tactile defensiveness via behavioral interventions (Wilbarger and Wilbarger 2002) can positively affect other lead-related deficits in children.

## Figures and Tables

**Table 1:** Correlations of blood lead concentration means with sensory processing scores.

Developmental period	Sensory magnitude	Sensory habituation
Very early (2–6 weeks)	0.39*	0.23
Early (2–12 weeks)	0.42**	0.25
Preweaning (14–26 weeks)	0.34*	0.13
6–12 months (28–52 weeks)	0.30#	0.03
Postchelation (68–112 weeks)	−0.23	0.26

*n* = 39, lead-exposed animals only.

* *p* < 0.05.

** *p* < 0.01.

# *p* < 0.10.

**Table 2:** Blood lead concentrations [mean ± SD (μg/dL)] for each treatment group at each time period.

	1-year lead		2-year lead	
Developmental period	No chelation	Chelation	No chelation	Chelation
Very early (2–6 weeks)	22.9 ± 11.0	24.5 ± 10.5	21.4 ± 5.8	27.1 ± 8.1
Early (2–12 weeks)	27.0 ± 9.2	29.6 ± 9.9	28.0 ± 6.6	30.6 ± 5.9
Preweaning (14–26 weeks)	36.4 ± 6.3	39.7 ± 6.5	37.5 ± 7.5	36.3 ± 7.8
6–12 months (28–52 weeks)	31.4 ± 3.2	31.6 ± 3.1	30.7 ± 6.8	32.6 ± 7.1
Postchelation (68–112 weeks)	11.4 ± 3.1	10.7 ± 3.8	40.1 ± 2.1	37.3 ± 3.6
